# With Great Age Comes Great Metastatic Ability: Ovarian Cancer and the Appeal of the Aging Peritoneal Microenvironment

**DOI:** 10.3390/cancers10070230

**Published:** 2018-07-10

**Authors:** Elizabeth I. Harper, Emma F. Sheedy, M. Sharon Stack

**Affiliations:** 1 Department of Chemistry and Biochemistry, University of Notre Dame, South Bend, IN 46617, USA; eharper1@nd.edu; 2 Harper Cancer Research Institute, University of Notre Dame, South Bend, IN 46617, USA; esheedy1@nd.edu; 3 Integrated Biomedical Sciences Program, University of Notre Dame, South Bend, IN 46617, USA; 4 Department of Mathematics, University of Notre Dame, South Bend, IN 46617, USA

**Keywords:** ovarian cancer, age, tumor microenvironment, extracellular matrix, mesothelial cells, immune, fibroblast, adipocytes, peritoneum

## Abstract

Age is one of the biggest risk factors for ovarian cancer. Older women have higher rates of diagnosis and death associated with the disease. In mouse models, it was shown that aged mice had greater tumor burden than their younger counterparts when intraperitoneally injected with ovarian tumor cells. While very few papers have been published looking at the direct link between ovarian cancer metastasis and age, there is a wealth of information on how age affects metastatic microenvironments. Mesothelial cells, the peritoneal extracellular matrix (ECM), fibroblasts, adipocytes and immune cells all exhibit distinct changes with age. The aged peritoneum hosts a higher number of senescent cells than its younger counterpart, in both the mesothelium and the stroma. These senescent cells promote an inflammatory profile and overexpress Matrix Metalloproteinases (MMPs), which remodel the ECM. The aged ECM is also modified by dysregulated collagen and laminin synthesis, increases in age-related crosslinking and increasing ovarian cancer invasion into the matrix. These changes contribute to a vastly different microenvironment in young and aged models for circulating ovarian cancer cells, creating a more welcoming “soil”.

## 1. Introduction

Ovarian cancer (OvCa) is the deadliest gynecological cancer, with a survival rate under 50% [1]. One of the biggest risk factors for OvCa is age, where the median age of diagnosis is 63 and median age of death is 70 [1]. Aging, as defined in the Hallmarks of Aging, is “the time-dependent functional decline that affects most living organisms” [2]. A call for research investigating the relationship between OvCa and aging was voiced in 1993 by Yancik after a review of epidemiologic data, showing older women were not only more likely to be diagnosed with OvCa but were more likely to die from their disease [3]. Yancik raised the question that has propelled the research in this field: why is there is a difference in survival between young and aged patients? Is there a difference in treatment, or does the cancer behave differently in older women? In 2013, epidemiological data were reviewed again by Trillsch et al. and their data suggest that older patients often receive less radical treatment, contributing to this disparity [4]. However, it is likely that there is more contributing to this disparity than physician partiality alone. A separate epidemiological study showed that older OvCa patients have a two-fold increase in peritoneal metastases relative to younger patients at time of diagnosis, suggesting that there is more to be discovered in the relationship between OvCa and aging [5]. Here we review the aging studies in the OvCa field, as well as aging studies involving distinct components of the peritoneal metastatic microenvironment.

OvCa metastasizes in a very unique fashion, where cells are exfoliated from the primary tumor as either single cells or multicellular aggregates and circulate through the peritoneal cavity via diffusion in the peritoneal fluid [6]. The circulating cells adhere to secondary sites, such as the omentum and parietal peritoneum, via interactions with mesothelial cells [6]. The OvCa cells induce mesothelial cell retraction, then invade into and anchor in the collagen-rich submesothelial matrix [6]. The OvCa cells can then proliferate and form a metastatic lesion [6]. Aging can affect nearly every step of this process.

**Figure 1:**
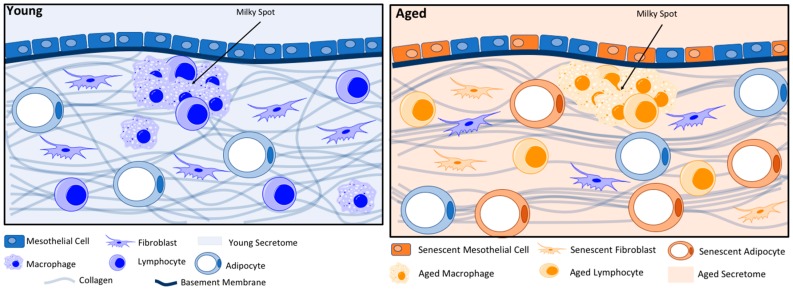
Changes in the Aged Microenvironment. Young: In the young metastatic microenvironment, collagens I and III form a directionally random meshwork that supports the tissue. In addition, there are low rates of senescence in mesothelial cells, fibroblasts and adipocytes, all of which secrete factors into the extracellular environment, forming the young secretome. The young secretome is characterized by decreased Matrix Metalloproteinase (MMP) expression, increased cytokine expression by immune cells, decreased cytokine expression by fibroblasts and decreased adipocyte-associated inflammatory factors. Milky spot immune cell aggregates exist in both young and aged metastatic environments, providing the tumor with abundant vascularization. Aged: In the aged metastatic microenvironment, there are lower levels of collagens I and III, which are remodeled to form more aligned, linear structures. In addition, higher levels of senescence alter the secretome, increasing inflammation and other factors that can promote ovarian cancer (OvCa) metastasis.

The peritoneum is a vast, serous membrane covering the interior of the abdomen and the visceral organs. The parietal peritoneum covers the interior of the abdominal wall, then folds to form the omentum, which lies between the parietal peritoneum and the anterior surface of the abdominal organs. The omentum is an organ rich in adipocytes and immune cells. Both the omentum and the parietal peritoneum are composed of a collagen-rich matrix covered by the mesothelium, separated by a thin basement membrane. The mesothelium is a single monolayer of simple squamous epithelial-like cells, or mesothelial cells, that cover the surface of the peritoneum. The basement membrane is a thin layer (<100 nm) composed mostly of collagen IV and laminin that separates the mesothelium from the elastic matrix below [7]. This matrix is comprised mostly of collagens I and III but contains other entities such as fibroblasts, immune cells, adipocytes, lymphatics and limited cardiovasculature [7]. Each of the components has the potential to react differently to OvCa cells through age-related changes (Figure 1).

The aging peritoneal microenvironment is defined in large part by two processes: extracellular matrix (ECM) remodeling and cellular senescence. Changes in collagen, laminin and fibronectin have the potential to alter how the metastatic OvCa cells invade into the peritoneum [8,9,10]. Senescence-induced changes in fibroblasts, mesothelial cells and immune cells drastically alter the secretome of the microenvironment, causing an increase in the transcription of factors that are associated with inflammation and angiogenesis [11,12]. Senescence is a cell’s permanent exit from the cell cycle and was first attributed to telomere attrition [13]. More recently, a number of factors have been identified that contribute to cellular senescence, including DNA damage [14,15], oxidative stress [16,17], high levels of glucose [18,19], transforming growth factor-β (TGF-β), [18,20,21,22] and the tumor suppressors p16^INK4a^ and p53 [2]. While senescence within the tumor itself suppresses tumor growth [2,23], senescence in the microenvironment has been shown to increase tumor growth [24,25].

Interestingly, the role of p53 also varies greatly between OvCa cells and microenvironment. It was reported that in a C57Bl/6 model, ID8 cells with a p53 deletion showed greater tumor growth than the ID8 parental cells [26]. However, p53 is overexpressed in the aging OvCa tumor microenvironment as a result of oxidative stress, oncogenic stress and DNA damage [27,28]. In response to severe damage, p53 determines cell fate, inducing either senescence or apoptosis. In epithelial and stromal cell lines, p53 more frequently induces senescence [28]. This leads to increased Matrix Metalloproteinase (MMP) secretion, a remodeling of the ECM and disruption of normal epithelial cell differentiation [28,29]. For reasons to be addressed, these effects contribute to increased OvCa metastasis and occur more dramatically in aged individuals.

## 2. Aging Modifies the Metastatic Microenvironment

*In vivo* models of intraperitoneal (IP) metastasis have been utilized to demonstrate an age-related difference in tumor burden in mice injected with ovarian tumor cells. When IP injected with syngeneic tumor cell lines, both C57Bl/6 and FVB mice exhibited a dramatic difference in disease progression between the young (3–6 months) and aged (20–23 months) cohort, with the aged mice harboring greater tumor burden than their younger counterparts [26]. Transcriptome analysis of gonadal adipose tissue from young and aged mice points to a difference in immune response in the aged mice but it is likely that the immune system is only one of the components of the microenvironment that is contributing to the age-related disparity in metastasis [26].

### 2.1. Mesothelial Cells

The mesothelium, a cobblestone monolayer of cells that exhibit characteristics of both epithelial and mesenchymal cells, lines the surface of the peritoneum. Its function in normal tissue is to create a barrier and limit the permeability of the peritoneum, as well as secretion of factors that are involved in peritoneal homeostasis and launching appropriate immune responses to pathogens [30]. These cells are very important in the adhesion of OvCa cells to secondary metastatic site. The senescent mesothelial population increases as the host ages, due to both increased rates of senescence as well as the resistance of senescent cells to pro-apoptotic signaling [5,31].

Senescent mesothelial cells change the cellular signaling in the tumor microenvironment, expressing factors such as fibronectin [16,32], intercellular adhesion molecule-1 (ICAM-1) [33], beta-galactosidase [31,34] and thymosin beta-10 [35]. Fibronectin, a mediator of cell-extracellular matrix interaction, has been shown to be increased in aging tissues [36]. This increase has been linked with increased OvCa cell adhesion [16] and increases tissue stiffness (which will be discussed in more detail in Section 2.3.1) [37]. The increase in OvCa cell adhesion is partially mediated by mesothelial ICAM-1, an adhesion molecule expressed by mesothelial cells that has been shown to be important in other abdominal cancers that metastasize to the peritoneum [33]. In addition, profiles of human peritoneal mesothelial cells isolated from young (mature adults under the age of 65) and aged (over the age of 65) patients showed an increase in inflammation-associated factors, suggesting increased inflammation in the aged mesothelium [38]. It was shown that age was associated with an increase in both the cyclooxygenase (COX) and nitric oxide synthase (NOX) pro-inflammatory systems, an upregulation of nuclear factor-κB (NF-κB) and inflammatory cytokines and an increase in reactive oxygen species (ROS) in mesothelial cells [38]. ROS have been shown to be a mediator of senescence; increased ROS results in increased cellular senescence [39]. Additional information on inflammation and the role of the immune system is included in Section 2.4.

Senescent mesothelial cells have been shown to interact with metastasizing OvCa cells, altering the OvCa secretome to express angiogenic agents such as chemokine CXC ligand 2 (CXCL1), chemokine CXC ligand 8 (CXCL8), hepatocyte growth factor (HGF) and vascular endothelial growth factor (VEGF) [40]. Mikuła-Pietrasik et al. saw increased angiogenesis in mouse models when OvCa was coinjected with senescent human peritoneal mesothelial cells (HPMCs) [40]. This process is mediated by TGF-β1 and IL-6, which are overexpressed in aged mesothelial cells [38,40]. When OvCa cells were incubated with senescent mesothelial cell conditioned media, they experienced higher levels of proliferation than those incubated with conditioned media from young cells, suggesting soluble factors released by senescent mesothelial cells promote the proliferation of OvCa cells [40]. OvCa cells with conditioned media from senescent mesothelial cells also showed greater migration and invasion *in vitro* [40]. In addition, histological analysis of patient tumors showed the presence of senescent mesothelial cells in cancerous tissues [40]. It is likely that an accumulation of senescent mesothelial cells, as seen in tissue from aged patients, provides a more welcoming metastatic niche for circulating OvCa cells [5].

Hyaluronic acid, or hyaluronan (HA), is a glycosaminoglycan secreted by cells with mesenchymal characteristics, such as mesothelial cells. It acts as a mediator of ECM organization as well as a lubricant on the mesothelial surface [41,42]. HA is also an FDA-approved treatment for osteoarthritis and is a popular treatment used by plastic surgeons to reverse the signs of aging of the skin [43,44]. Relevant studies have shown two divergent lines of research: HA increasing [45,46,47] or decreasing [48,49,50] cell adhesion. However, certain OvCa cells lines have been shown to bind directly to HA, suggesting HA increases OvCa cell adhesion [46]. This likely contributes to the observation in ovarian and prostate cancer patients, where overexpression of HA generally results in a poorer prognosis [11,51]. In addition, HA has been shown to impact cell migration [52,53,54] and cell proliferation [54,55,56], to activate skin fibroblasts [57] and to be upregulated in response to inflammation [58]. There is not much information available on the effects of age on HA other than an observed decrease in aged tissue, likely due to the decreased synthetic capacity of aged cells [41,59]. However, the use of HA in the treatment of age-related diseases suggests that the role of HA in the aging microenvironment warrants further investigation.

### 2.2. Extracellular Matrix

The peritoneal ECM is a complex system that supports the cells of the peritoneum. Made up of collagen, laminin and fibronection, the ECM plays an integral role in both normal peritoneal structure as well as the metastatic success of OvCa. Directly beneath the mesothelial layer is a thin basement membrane composed of collagen IV and laminin, covering an elastic matrix of collagens I and III, laminin and fibronectin [60]. The ECM changes drastically with age, which can change how integrins and syndecans bind to the ECM, thus altering the interaction between the metastasizing OvCa cells and the tumor microenvironment [60], including increased adhesion of macrophages [61] and increased cancer cell invasion [62].

#### 2.2.1. Collagen

Collagen is one of the most abundant proteins in the body and forms a large portion of the peritoneal extracellular matrix. There are multiple types of collagens; in the context of the peritoneum, collagens I and III are the most notable, both of which are fibrous collagens [60]. On a molecular level, both I and III have a similar amino acid structure distinct from other proteins, with glycine repeating every third amino acid and a high percentage of prolines, which are often post-translationally modified to become hydroxyprolines [63]. These amino acids chains come together to form the characteristic triple helix, which are banded together in an overlapping manner to form fibrils with the distinct D-banding pattern [63].

**Figure 2:**
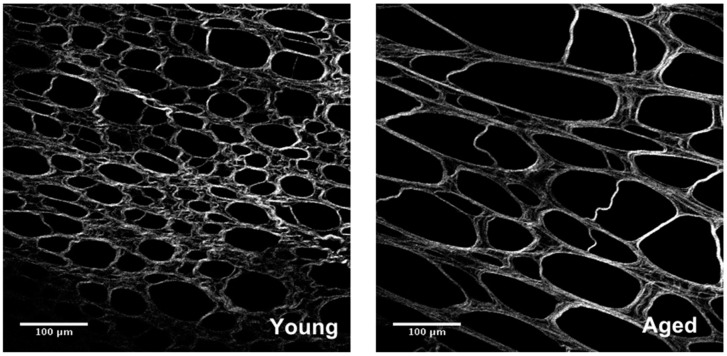
Age-related changes in omental collagen structure. Second harmonic generation imaging of omental tissue isolated from young and aged mice shows a distinct difference in structure. Aged collagen forms crosslinks that result in the loss of meshwork, formation of tendon-like structures and increased anisotropy. This causes a disruption of tissue structure that can affect how metastasizing OvCa cells interact with the tumor microenvironment.

While little research has been done on the effects of aging on peritoneal collagen, there is a wealth of information on skin collagen. As far back as 1975, scientists noted a significant decrease in the amount of collagen in aged skin [64]. An immunohistochemical analysis showed amounts of collagens I and III change as an individual ages [65]. Both collagens decrease in aged tissue but the ratio of collagen I/collagen III increases, suggesting that collagen I is decreasing at a slower rate than collagen III [65]. The structure of collagen is disrupted with age, resulting in disorganization of the fibers (Figure 2) [11]. In addition to skin, collagen extracted from human arteries, mouse tails and mouse prostates showed alterations not only in structure but also in mRNA and protein expression, pointing to a decrease in collagen synthesis as the culprit behind the decreasing amounts of collagen [11,66,67]. Later research showed that this decrease is likely due not only to decreased synthesis but increased degradation as well [11,68].

Matrix Metalloproteinases, or MMPs, are the main source of ECM degradation [69]. MMPs are a family of 23 zinc-dependent enzymes that are divided into distinct groups: collagenases, gelatinases, matrilysins and membrane-type MMPs [69]. Outside of gene regulation, MMPs are regulated in two major ways: they require activation from the zymogen form in order to be active and active MMPs are regulated by tissue inhibitors of metalloproteinases, or TIMPs [69,70]. MMPs are secreted from numerous cell types within the microenvironment, such as fibroblasts and immune cells, as well as the OvCa cells themselves [69,71]. Three MMPs have been shown to be upregulated in OvCa: MMP2, MMP9 and MMP14 (also referred to as MT-MMP1) [13]. MMP14, a membrane-type, is present at high levels in the tumor cells themselves, while MMP9, a gelatinase specific to collagens IV and V, is more often upregulated in the stroma [71]. In addition, senescent cells have been shown to have an increased expression of MMPs and addition of an MMP inhibitor reverses some senescent-specific tissue phenotypes [72]. An upregulation of either MMP9 or MMP14 in the stroma around the tumor cells is correlated with a more invasive phenotype, pointing to a critical role of MMPs in the tumor microenvironment [71].

Due to its long half-life, post-translational modifications accumulate in collagenous tissue over time [73]. Of particular interest here are modifications that create covalent crosslinks between collagen molecules. Lysyl oxidase, or LOX, is a family of enzymes that modify lysine sidechains to form desmosine through a Schiff base intermediate [74]. Recently, increased LOX crosslinks have been shown to play a role in chemoresistance [75]. Advanced Glycation End-products, or AGEs, are formed non-enzymatically as a result of glycosylation over time. These crosslinks have been shown to change the structure and mechanical properties of collagen-rich tissues, such as the peritoneum [76]. Crosslinked collagen has higher fiber alignment, resulting in more tendon-like structures, causing the tissue to lose elasticity and become stiffer than non-crosslinked collagen (Figure 2) [76,77]. An increase in AGEs has been correlated with increased peritoneal permeability, which could contribute to increased OvCa invasion [8]. In addition, stiffer matrices have been shown to increase cell motility, proliferation and adhesion [9,10].

When AGEs occur in serum albumin, they can bind to the AGE receptor (RAGE) on monocytes and trigger the release of tumor necrosis factor-α (TNF-α), leading to insulin resistance [78]. When bound to RAGE on adipocytes, AGEs can induce the formation of ROS [78]. As mentioned in Section 2.1, ROS have been shown to be a mediator of cellular senescence, where high levels cause enough cellular damage for the cells to leave the cell cycle permanently [39]. Additionally, ROS can activate p53, which is another pathway leading to cellular senescence [39]. In addition to their role in changing the structure of the ECM, AGEs can also induce senescence in numerous cell types in the microenvironment through formation of ROS and subsequent pathways [39,78].

#### 2.2.2. Fibronectin

In contrast to the helical nature of collagen, fibronectin is a structural glycoprotein that forms repeating beta-sheets in its folded form [37,79]. One of the main roles of fibronectin is mediating cell-cell interactions [36]. Not only does the amount of fibronectin increase in aged tissues but aging fibronectin, like collagen, shows an increase in anisotropy with age [36,37,79]. Fibronectin has also been shown to stretch with age, resulting in increased stiffness [37]. In addition, fibroblasts interacting with aged fibronectin responded differently than when interacting with young fibronectin [37]. The fibroblasts interacting with aged fibronectin were shown to have longer β1 integrin adhesions as well as more actin stress fibers [37]. In addition, as mentioned in Section 2.1, senescent mesothelial cells express more fibronection, contributing to increased OvCa cell adhesion mediated by the α5β1 integrin [16].

#### 2.2.3. Basement Membrane

The basement membrane (BM) is a component of the extracellular matrix that separates epithelial cells from underlying connective tissue. It is primarily composed of collagen IV, intertwined with laminin polymers [80,81]. The BM exhibits structural changes as it ages, most notably with aged cells synthesizing less collagen IV than young cells [82,83]. While the basement membrane is understood to thicken with age, the declined synthesis of collagen IV indicates that the thickness is due to decreased turnover of aged tissues [82,83].

In primary ovarian tumors, collagen IV is absent on the ovarian surface [84]. This indicates that OvCa cells must firstly degrade the ovarian BM (specifically, degrading collagen IV) to detach from the ovary and shed into the intraperitoneal space [84]. Following this migration, cells then alter the mesothelial BM to anchor and proliferate [85]. The mesothelial BM also has high collagen IV and laminin content [85], so OvCa cells must again degrade collagen IV to gain entry into the underlying ECM.

Disabled-2 (Dab2) is a signal transduction protein and tumor suppressor that also functions in positional organization of ovarian surface cells. In OvCa, genetic and epigenetic changes to Dab2 enable tumor cells to escape ovarian BM control and proliferate in a disorganized fashion, resulting in diffusion into the peritoneal cavity and metastasis [84]. Hypermethylation of the Dab2 promoter results in epigenetic silencing of the gene, which is correlated with a loss of expression of collagen IV [86]. Methylation patterns are known to change with age [87] and the effects of aging on methylation can vary from inducing DNA hypomethylation to inducing hypermethylation. Such age-associated deviation in methylation leads to advanced epigenetic damage in aged individuals [88]. It is possible that DNA hyper-methylation of the Dab2 promoter may be affected by age, thereby impeding collagen IV expression—increasing BM susceptibility to degradation.

OvCa cells first bind to mesothelial cells to gain entry into the underlying matrix [85]. This adhesion is facilitated by ovarian cancer antigen CA125 and mesothelin interaction [89], and/or by integrins such as β_1_-integrin and cell surface receptors such as CD44 (the receptor for HA) [85]. Upon attachment to the mesothelium, OvCa cells upregulate MMP production, including that of MMP2 [85]. MMP2 preferentially interacts with collagen IV, resulting in the loss of basement membrane [90]. As aged cells are downregulated in their expression of collagen IV [83], this may lead to more efficient BM degradation in the aged host. Additionally, in many cancers, Dab2 downregulation leads to increased transcription of the ribonucleoprotein hnRNPK, which then enhances MMP2 transcription by the metastatic cells [91]. Thus, downregulation of Dab2, as observed in OvCa metastatic cell lines, may be correlated with increased MMP2 expression.

In addition to collagen IV, laminin provides structural support in the basement membrane [11,92]. Laminin is a trimeric protein with high homology between the alpha, beta and gamma trimers [11,92]. It is highly regulated in adults; the biggest changes observed in aging studies are the replacement of fetal laminin with adult laminin [11,93]. However, in carcinogenesis, it was observed that prostate tissues experience a loss of adult laminin, which results in disorganization of epithelial cells [11,93]. In addition, some tumor cells have been shown to increase expression of laminins, increasing cell adhesion and invasion [92]. In the context of aging, it has been shown that there are decreased levels of laminin in aged basement membranes [94,95]. In addition, laminins can also be AGE-modified, leading to decreased laminin-collagen IV binding, which may make it easier for the OvCa cells to invade through the basement membrane [94]. AGE modifications have also been shown to increase laminin synthesis, however they also impair laminin assembly, likely contributing to the described decrease in total laminin [94].

### 2.3. Fibroblasts

#### 2.3.1. Senescent Fibroblasts

**Figure 3:**
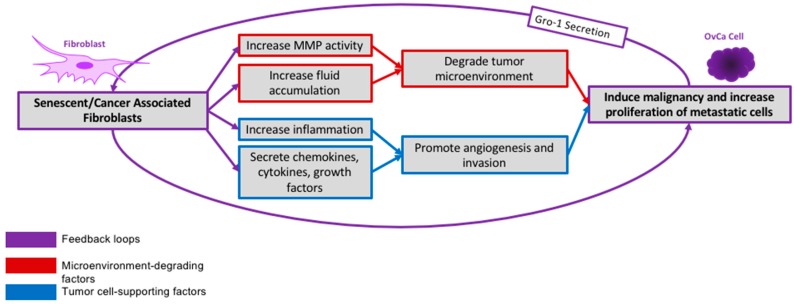
Stromal-Epithelial Crosstalk. Active crosstalk occurs between senescent and activated fibroblasts and OvCa cells. This induces activated fibroblasts, while concurrently inducing proliferation and malignancy of the invading tumor.

Fibroblasts are a stromal cell type, functioning in upkeep of the connective tissue environment and ECM [96]. This upkeep is greatly altered with age in ways that promote tumorigenesis, such as increasing angiogenesis and stimulating OvCa cell growth [97,98]. Aged fibroblasts secrete less collagen and other proteins than their younger counterparts [96]. Furthermore, fibroblasts isolated from older individuals had far higher rates of senescence than fibroblasts from younger individuals with age, senescent fibroblasts accrue and replace presenescent cells (Figure 3) [24,25,96,99]—greatly altering the function of the tissue in the process. Notably, accumulation of senescent fibroblasts in the OvCa microenvironment is associated with increased cell proliferation and metastatic potential due to interactions with the cancer cells [24].

In a murine model, senescent fibroblasts partake in significant stromal-epithelial crosstalk (Figure 3) [100], inducing premalignant epithelial cells to lose differentiation capacity, increase invasiveness and eventually become fully malignant cells [99]. This can be attributed to many factors secreted by senescent fibroblasts that alter the tissue microenvironment and stimulate growth of epithelial cells expressing oncogenic mutations [12,101,102]. Increased fibroblast senescence results in greater secretion of vascular endothelial growth factor (VEGF), which increases angiogenesis of the region [97,98]. As tumors necessitate a vascular supply for efficient growth [103], increased angiogenesis supports epithelial tumor growth. Senescent fibroblasts also secrete more MMPs [102], which degrade collagen and the basement membrane [69,90]. These effects have been widely shown to be correlated with increased cancer cell growth (Figure 3) [71,84]. Secretion of MMPs by senescent fibroblasts also results in heightened microvascular permeability leading to a buildup of extracellular fluid, which increases inflammation and damages the surrounding tissue matrix, possibly altering the natural anti-tumorigenic nature of the presenescent microenvironment [72].

#### 2.3.2. Fibroblast Activation

Epithelial tumor cells activate fibroblasts in the tumor stroma, stimulating a phenotypic switch from normal fibroblasts to cancer-associated fibroblasts (CAFs) [104]. Epithelial OvCa cells secrete factors such as chemokine growth-regulated oncogene 1 (Gro-1) [100]. Gro-1 induces the CAF phenotype and, as Gro-1 is overexpressed in OvCa patients, there is significant evidence that Gro-1 alters the stromal environment to induce senescence in fibroblasts. This epithelial-stromal interaction is critical in tumor initiation and proliferation. Ovarian CAFs promote tumor growth by secreting cytokines and chemokines into the microenvironment [98,104], while non-recruited, presenescent fibroblasts do not enhance tumorigenesis [100].

CAFs exhibit many of the same general characteristics as senescent fibroblasts [25,102]. Thus, as OvCa cells recruit CAFs, they also induce pro-tumorigenic microenvironment changes as caused by senescent fibroblasts described above (Figure 3). Both senescent fibroblasts and CAFs secrete CXCL12 [105], among other pertinent factors such as IL6, IL8 and MMPs [106]. These increase inflammation and promote angiogenesis, invasiveness and metastasis [105,106]. Secretion of chemokines—as observed in both CAFs and senescent fibroblasts—is likely a key cancer-promoting function of fibroblasts [105]. Thus, aging and increased senescence of fibroblasts alter the microenvironment and oncogenic cells themselves in a way highly conducive to tumor growth (Figure 3).

### 2.4. Immune Cells

#### 2.4.1. Tumor Cells Preferentially Adhere to Immune Cell Clusters

Ovarian cancer cells shed from the primary tumor and adhere preferentially to the peritoneum or omentum in the abdominal cavity. The omentum, a visceral adipose tissue, is known to have a large influence on peritoneal immunity due to its high quantity of lymphoid aggregates (Figure 1), often called milky spots [107]. Within the omentum, initial attachment and growth of tumors were observed to be most prevalent surrounding organized aggregates of immune cells [108]. Omental stem cells exhibit a large capacity to produce angiogenic growth factors, resulting in high vascularization of the region, particularly surrounding immune cells [103]. Avascular tumors are severely limited in growth due to a lack of blood supply. Tumors must make an “angiogenic switch” to proliferate, where the initial metastatic tumor initiates the formation of new vessels for increased blood supply [109]. However, the tumor must anchor to a membrane before it can make the angiogenic switch. Studies show that tumor cells preferentially bind to mesothelial cells directly above the omental immune cell cluster, where the initial tumor is provided with an abundant blood supply from the existing vasculature of the immune cell cluster. This contributes to the high survival rate of metastatic cells in the omentum [108].

Intraperitoneal injection of green fluorescent protein (GFP)-expressing tumor cells showed localization to milky spots in the omentum [108]. This supports prior conclusions that migration and attachment of tumor cells to the omentum and specifically to immune aggregates, occurs from migration from the peritoneal cavity and does not necessitate intravascular transportation. As tumor cells metastasize, they disturb the structure of the immune cell aggregate and eventually displace all immune cells from the metastatic tumor mass [108]. It is important to note, however, that while hematogenous metastasis of OvCa to the peritoneal and omentum is not critical for cancer spread, intravascular transportation of the tumor does occur with significant metastatic results [110,111].

#### 2.4.2. Aging Affects Antitumor Macrophage Function in Peritoneum

Milky spot aggregates are comprised primarily of macrophages [112]. Studies exploring the effect of aging on macrophage function prior to tumor exposure have yielded conflicting results, although such discrepancies could be due to differences in sex, strain, species, or site of tissue in macrophage isolation [113]. Isolated macrophages specifically from the peritoneum indicate lower levels of inflammatory cytokine production with age (Table 1) [113,114]. Particularly, replicated *in vitro* results indicate that macrophages of young mice produce higher amounts of tumor necrosis factor-α (TNF-α), MMPs and have a higher phagocytic capacity than aged mice (Table 1) [115,116,117].

**Figure 4:**
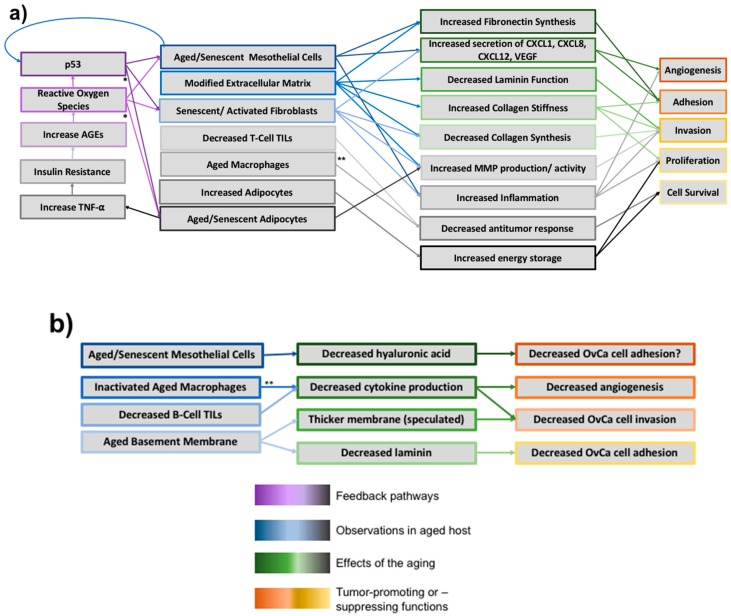
The effects of aging on the peritoneal microenvironment. (**a**) Tumor-Inducing Effects: Aging of the host stimulates a vast and interconnected network of alterations to the peritoneal microenvironment. These changes are often correlated with increased tumor burden due to heightened angiogenesis of the region and OvCa cell adhesion, invasion, proliferation and survival. As depicted, the multifactorial causes and results of aging present significant challenges for analysis; (**b**) Tumor-Suppressing Effects: While our review generally concludes that aging alters the microenvironment in a way conducive to tumor growth, in contrast certain aspects of aging seem to impair tumorigenesis. Aged and senescent mesothelial cells secrete less hyaluronic acid, which is hypothesized to decrease OvCa adhesion to the extracellular matrix (ECM). Inactivated aged macrophages are less capable of cytokine secretion, which thereby decreases angiogenesis potential and cell invasion. The aged BM thickens due to less collagen IV turnover, which we speculate could in theory decrease OvCa invasion (however, to our knowledge no conclusions have been drawn regarding this). The aged basement membrane (BM) also has a decreased laminin content, which may decrease cell adhesion. * While not shown to be a causative link, in aged adipose tissue there is an increase in reactive oxygen species (ROS) that is correlated with adipocytes presenting a senescent phenotype, suggesting that ROS plays the same role in adipocytes that it does in other cell types [145]. p53 has been shown to have numerous effects on adipose tissue and is likely also contributing to the senescent phenotype [145]. ** Aged macrophages paradigm: aged macrophages have been shown to both induce tumorigenesis and inhibit it., we depict both pathways. Note: Color gradients intended to help viewer differentiate between different effects of each component of the aging microenvironment.

Studying the effect of decreased cytokine secretion on cancer in models of aging yields highly conflicting results (Table 1, Figure 4). Firstly, TNF-α has both pro- and anti-tumorigenic effects. On one hand, TNF-α could promote cancer due to its activation of cancer-promoting pathways such as NF-κB and its correlation with increased angiogenesis, cell growth and metastasis [106,118]. On the other hand, TNF-α also has inherent anti-tumor effects: the cytokine activates tumor-infiltrating dendritic cells and promotes tumor stroma destruction [106,119]. Phagocytic efficiency and general cytotoxic capabilities also decrease in aged models [120]. This could lead to increased cell proliferation in the aged host, a hallmark of cancer [121]. However, increased cytokine secretion can lead to increased inflammation in the tissue. This results in a mutagenic microenvironment abundant in growth factors and cytokines that sustain angiogenesis, proliferation and invasion [122], which are three other hallmarks [121]. It is difficult to conclude whether the anti-tumor killing abilities of the aged macrophage outweigh its inability to provide support to the tumor (Table 1).

Notably, the innate immune response of macrophages is affected by their environment [113,115]. Peritoneal macrophage function, including cytokine secretion, was observed to be altered with age only due to changes in the aged microenvironment, not inherent age-related dysfunction of the macrophage itself [115]. Thus, it is possible to restore the macrophage to its full secretory phenotype by changing its environment [113,115]. Epithelial cancer cells and stromal cells do just this–they secrete growth factors and cytokines such as macrophage colony-stimulating factor 1 (CSF-1) to recruit macrophages, converting their phenotype into tumor-associated macrophages (TAMs) [121,123,124]. When activated, TAMs work similarly to cancer associated fibroblasts (CAFs). They promote metastasis by secreting growth factors and cytokines by increasing angiogenesis and participating in cross-talk with epithelial cells and stromal cells [121,124]. Increased CSF1 density and increased TAM occurrence are correlated with decreased survival rates [121,124]. However, it is not understood whether cytokine secretion is downregulated in aged TAMs, as occurs in pre-activated macrophages.

#### 2.4.3. Tumor Infiltrating Lymphocytes: B and T cells

T-cell associated tumor infiltrating lymphocytes (TILs) are correlated with increased survival in OvCa patients (Table 1). CD4+ and CD8+ T-lymphocytes are two types of TILs which recognize cancer antigens and inhibit cancer proliferation. CD4+ TILs elicit dendritic cell responses, which then induce CD8+ cells to provide extended cytotoxicity, killing tumor cells (Figure 4). Thus, an increase in CD4+ and CD8+ T-lymphocytes is a survival advantage in OvCa patients [121,125]. One factor in this pathway is IL-2 secretion: increased IL-2 secretion results in activated macrophages and tumor lysis directly from CD8+ T-lymphocytes [125].

T-cell production and function is widely known to decrease with age. Notably, aged CD4+ T-cells experience higher degrees of apoptosis and decreased function when compared to young T-cells in an aged murine model. Aged CD4+ T-cells showed less expression of CD4 and a lower mitochondrial mass [126]. Furthermore, aged CD4+ T-cells secreted less IL-2 than young phenotypes [127] and have decreased memory capabilities [128]. These factors indicate that aged CD4+ TILs are inherently less active than young TILs and therefore express less antitumorigenic capacity (Table 1).

The effect of B-cell TIL function on OvCa presents more difficult data (Table 1). In some studies, B-cell TILs, such as CD20+, are also understood to bear a tumor survival advantage in OvCa patients [125,129]. Studies showed that using anti-CD20+ antibodies in B-cells result in decreased CD8+ antitumor functionality, which links B-cell advantage to that of CD8+ T-cells. A lack of CD20+ secretion results in decreased CD8+ cytotoxic capabilities, promoting cancer development [130]. While CD8+ T-cells function in antitumor activity on their own, effectiveness is shown to increase in the presence of CD20+ [129]. Similar to T-cells, aged B-cells exhibit decreased antibody affinity and memory responses [131]. Consequently, aging downregulates the CD20+ and CD8+ association, resulting in decreased tumor lysis and poorer OvCa prognosis (Table 1). However, reports of certain aged B-cells such as B1a lose many immunosuppressive functions with age but notably gain the capacity to stimulate T-cell CD8+ tumor-killing activity [132]. Other reports on OvCa, also present data that increased B-cell inflammatory activity in ovarian tumors is associated with poorer prognoses [133,134]. Certain populations of B-cells, such as CD138+, instead increase angiogenesis and disrupt the T-cell lymphocyte antitumor response. Reports show reduced survival of individuals with ovarian tumors presenting high CD138+ B-cell counts, possibly due to tumor-induced alterations of B-cell phenotype [133]. Studies of OvCa patients also conclude that higher numbers of CD19+ B-cells are correlated with increased tumor severity [134]. High B-cell activity is a trait generally attributed to a younger individual [131] and thus the conflicting results of B-cell TIL contribution to OvCa proliferation cannot be fully resolved by literature results.

### 2.5. Adipocytes

Adipocytes make up the majority of the omentum and are present throughout the peritoneum [6]. Adipocytes are a complicated cell group that play a very important role in metabolism. In addition, adipocytes fuel OvCa metastatic success by providing energy in the form of fatty acids and lipids [135]. In addition to this role, adipocytes have been shown to secrete IL-8 and adipokines, which help guide OvCa cells to metastatic sites [135,136]. It has been shown that body fat percentage increases with age, as well as the capability of adipocytes to migrate out of their normal adipose tissues and into other sites of the body, causing site-specific alterations [136,137,138]. Specifically, aged adipocytes migrate to the viscera in the abdominal cavity, which is linked with higher disease rates than fat depots in other areas [137,139]. In fact, surgical removal of visceral fat in rats alleviated obesity-related symptoms, such as metabolic disease and insulin resistance and lengthened the lifespan of the rats [139,140,141]. Epidemiologic data show that obesity is a risk factor for worse disease in women, notably age-related diseases such as OvCa [136,142]. An *in vivo* pre-clinical study showed that obese mice intraperitoneally injected with OvCa cells (either diet-induced obesity or leptin-mutant) have an increased tumor burden over their lean counterparts [143]. Recently, there has been a surge of research on aging adipocytes; it has even been suggested that obesity accelerates aging, or that aging- and obesity-related processes mirror each other [136].

Aging adipocytes have been correlated with chronic inflammation [136]. Adipose tissue macrophages, or ATMs, have been shown to increase with age [136]. These immune cells secrete IL-6, promoting inflammation [136]. In addition, aged adipose tissue has higher rates of cellular senescence, as seen in the other cell types mentioned in previous sections [136]. These senescent cells also promote inflammation through the secretion of factors such as chemokines, cytokines, growth factors and MMPs [136]. In addition, the amount of differentiated and mature adipocytes formed from preadipocytes decrease with age, increasing the percentage of preadipocytes in aged tissue [137]. These preadipocytes secrete a proinflammatory profile similar to senescent cells, with factors such as PAI, IL-6 and proinflammatory cytokines and chemokines [137,144].

In addition to inflammation, aging adipocytes have been correlated with insulin resistance [136,145]. AGE modifications on serum albumin have been shown to cause an increase in ROS in adipocytes, which blocks cell differentiation and leads to insulin resistance [78]. AGEs prevent cellular uptake of glucose, which can raise glucose levels, potentially contributing to AGE-mediated collagen crosslinks (see Section 2.2.1) [78,146]. Serum-AGE levels were shown to be higher in aged mice versus young, contributing to more ROS and less glucose uptake [78]. In addition, serum AGEs have been shown to stimulate TNF-α in monocytes, which causes insulin resistance [145,147].

## 3. Conclusions

While this review has divided the peritoneal microenvironment into distinct cellular or functional units, in reality there is complex crosstalk between all components of the microenvironment that is just beginning to be uncovered and understood. The end result is a vastly different metastatic microenvironment in aged patients relative to that seen in young patients (Figure 1), reminiscent of one of the first big debates in the field: the seed-and-soil hypothesis. Based on the research discussed above, it is clear that the aging peritoneum provides a better “soil” for metastasizing OvCa cells. Each component of the microenvironment has the potential to affect OvCa metastasis in a variety of ways (Figure 4).

At every step of the establishment of metastases, we see differences in aged hosts. OvCa cells first adhere to mesothelial cells; aged hosts have higher numbers of senescent mesothelial cells, which increase inflammation and also increase factors such as fibronectin and ICAM-1 that mediate cell-cell adhesion [16,33,38]. Once the OvCa cells adhere to and disrupt the mesothelial cells, they next invade into the collagen-rich matrix below. Aged hosts have an increase in MMP activity and lower rates of collagen synthesis, resulting in a less dense matrix that facilitates invasion. In addition, aged collagen accumulates crosslinks, which make the tissue stiffer and more aligned, allowing OvCa cells to adhere more readily [8,75]. The other cells present in the microenvironment, including fibroblasts, immune cells and adipocytes, also play a large role in changing the metastatic microenvironment. Aged fibroblasts secrete less collagen than their younger counterparts and senescent fibroblasts share many of the characteristics of CAFs, promoting OvCa metastasis [24,96]. In the immune landscape, it is unclear whether the effect of age on macrophages promotes or obstructs tumor growth. However, it can be concluded T-cell lymphocytes and certain B-cell lymphocytes experience a loss of function with age, resulting in less regulated tumor growth and increased proliferation [125]. Aged individuals have been shown to have increased adipocyte deposits, which provide energy for the OvCa metastases [137]. Aged adipose tissue also has a chronic inflammation response, resulting in immune stimulation as well as secretion of elements such as growth factors and MMPs, that can contribute to OvCa invasion and proliferation [136]. These molecular processes may also represent targets for therapeutic intervention in the aged host.

There are not many therapeutic interventions that target aging. Recent studies of senescence and the senescent-associated secretory phenotype (SASP) illuminate the field of senolytics as a promising anti-cancer treatment [148,149]. Many senolytic drugs have been discovered and tested in murine models, working to selectively target the senescent cells’ anti-apoptotic pathways to induce cell death [148]. In murine models, this decreases the SASP to decrease cancer spread [148]. Notably, this is a selective treatment [148,149]—not every senescent cell has to be eliminated. Much work remains to bring this field to clinical trial stages but this review supports the observation that senolytic treatments are a propitious focus for age-associated cancers.

The studies performed in this field to date have shown that aging has multi-faceted effects on the tumor microenvironment. However, many questions remain. Much of the work reviewed here is not specific to the peritoneal tumor microenvironment and many studies were performed outside the context of OvCa metastasis. Just as Yancik voiced in 1993, there is still a need for aging research in the OvCa field. As the field progresses, integrating research on the molecular mechanisms of aging may reveal new targets for anti-metastatic therapies for OvCa patients.

## Figures and Tables

**Table 1:** Summary of aging-related immune changes.

Immune Cell Component	Effect of Aging	Effect on OvCa Metastasis
**T-cell Tumor Infiltrating Lymphocytes**	Decreased cytokine secretion Increased apoptosis Decreased lymphocyte association	Decreased tumor lysis leads to increased proliferation
**B-Cell tumor Infiltrating Lymphocytes**	Decreased cytokine secretion Decreased T-cell association	Possibly increased angiogenesis, possibly decreased tumor lysis
**Pre-Activated Macrophages**	Decreased cytokine secretion Decreased phagocytic activity	Unknown, possibly mixed effects.
